# Pegylated-Interferon Alpha Therapy for Treatment-Experienced Chronic Hepatitis B Patients

**DOI:** 10.1371/journal.pone.0122259

**Published:** 2015-04-02

**Authors:** Ming-Lun Yeh, Cheng-Yuan Peng, Chia-Yen Dai, Hsueh-Chou Lai, Chung-Feng Huang, Ming-Yen Hsieh, Jee-Fu Huang, Shinn-Cherng Chen, Zu-Yau Lin, Ming-Lung Yu, Wan-Long Chuang

**Affiliations:** 1 Division of Hepatobiliary, Department of Internal Medicine, Kaohsiung Medical University Hospital, Kaohsiung, Taiwan; 2 Division of Hepatogastroenterology, Department of Internal Medicine, China Medical University Hospital, Taichung, Taiwan; 3 Graduate Institute of Medicine, College of Medicine, Kaohsiung Medical University, Kaohsiung, Taiwan; 4 Faculty of Internal Medicine, College of Medicine, Kaohsiung Medical University, Kaohsiung, Taiwan; 5 Department of Occupational Medicine, Kaohsiung Municipal Ta-Tung Hospital, Kaohsiung Medical University, Kaohsiung, Taiwan; 6 Departmentof Internal Medicine, Kaohsiung Municipal Hsiao-Kang Hospital, Kaohsiung Medical University, Kaohsiung, Taiwan; 7 Departmentof Internal Medicine, Kaohsiung Municipal Ta-Tung Hospital, Kaohsiung Medical University, Kaohsiung, Taiwan; 8 Graduate Institute of Clinical Medicine, College of Medicine, Kaohsiung Medical University, Kaohsiung, Taiwan; Centers for Disease Control and Prevention, UNITED STATES

## Abstract

**Background:**

Studies are limited on pegylated interferon (Peg-IFN) therapy for chronic hepatitis B (CHB) patients who failed or relapsed on previous antiviral therapy.

**Objectives:**

We aimed to investigate the effect of Peg-IFN therapy in treatment-experienced CHB patients.

**Study Design:**

A total of 57 treatment-experienced CHB patients at two medical centers were enrolled. All of the patients were treated with Peg-IFN α-2a at 180 μg weekly for 24 or 48 weeks. The hepatitis B serological markers and viral loads were tested every 3 months until 1 year after stopping Peg-IFN therapy. The endpoints were HBV DNA <2000IU/mL, hepatitis B e antigen (HBeAg) seroconversion, and a hepatitis B surface antigen (HBsAg) loss at 12 months post-treatment.

**Results:**

In HBeAg-positive patients, 25.0%, 29.2%, and 12.5% of the patients achieved HBeAg seroconversion, HBV DNA <2000 IU/mL and a combined response, respectively, at 12 months post-treatment. Prior IFN therapy, a high baseline ALT level, a low creatinine level, undetectable HBV DNA at 12 weeks and a decline in HBV DNA >2 log_10_ IU/mL at 12 weeks of therapy were factors associated with treatment response. In HBeAg-negative patients, 9.1%, 15.2%, and 6.1% of the patients achieved undetectable HBV DNA, HBV DNA <2000 IU/mL, and an HBsAg loss, respectively, at 12 months post-treatment. No factor was significantly associated with the treatment response in the HBeAg-negative patients. The median HBsAg level declined from 3.4 to 2.6 log_10_ IU/mL in all the patients, and the 5-year cumulative rate of the HBsAg loss was 9.8% in the HBeAg-negative patients. Overall, none of the patients prematurely discontinued the Peg-IFN therapy.

**Conclusions:**

Peg-IFN re-treatment is effective for a proportion of HBeAg-positive treatment-experienced patients; it has limited efficacy for HBeAg-negative treatment-experienced patients. Peg-IFN might facilitate HBsAg loss in HBeAg-negative treatment-experienced patients.

## Introduction

Chronic hepatitis B (CHB) is a global health problem, with an estimated 350 million people being chronically infected.[1] CHB is the most common cause of chronic liver disease and hepatocellular carcinoma (HCC) in Asia.[2] Approximately 15–40% of CHB patients eventually develop cirrhosis, hepatic failure and HCC. A reduced risk of hepatic decompensation and HCC as well as a reversion of hepatic fibrosis by long-term anti-HBV therapy have recently been demonstrated.[3–6]

The current recommended first-line antiviral therapies for CHB include pegylated-interferon (Peg-IFN) α-2a, entecavir (ETV) and tenofovir disoproxil fumarate (TDF). Compared with nucleos(t)ide analogue (NUC) therapy, Peg-IFN therapy has the advantages of a finite duration, an absence of resistance and higher rates of anti-HBe seroconversion with 12 months of therapy. Peg-IFN therapy has the disadvantages of having a moderate antiviral effect, inferior tolerability and a risk of adverse events. Although Peg-IFN therapy has higher rates of anti-HBe seroconversion with 12 months of therapy, the efficacy is unsatisfactory. For Hepatitis B e antigen (HBeAg)-positive patients, one year of Peg-IFN therapy achieved a 22–27% HBeAg seroconversion rate at the end of treatment, which increased to 35% 1–2 years after stopping treatment.[7] For HBeAg-negative patients, one year of Peg-IFN therapy achieved a 63% undetectable HBV DNA rate; the rate rapidly dropped to 15% at 1 year after stopping treatment.[8] Although NUC therapy has the advantage of having a potent antiviral effect, poor durability of the effectiveness after stopping NUC therapy is encountered in the majority of patients. In a study by Reijnders J.G. et al., over 40% and 50% of HBeAg-positive patients experienced HBeAg seroreversion and recurrent viremia, respectively, 4 years after HBeAg seroconversion.[9] One-year and 3-year relapse rates of 44% and 52%, respectively, were reported in HBeAg-negative patients after lamivudine therapy was stopped.[10] Another recent study investigating the off-therapy durability of ETV in HBeAg-negative CHB patients showed that 45.3% of the 95 patients had a clinical relapse within 1-year after stopping ETV therapy.[11]

Re-treatment of CHB is a continuing concern in a substantial proportion of patients after antiviral therapy. Limited studies have been reported on Peg-IFN treatment for patients who failed or relapsed on previous antiviral therapy. We aimed to investigate the efficacy of Peg-IFN therapy in treatment-experienced CHB patients and the factors associated with treatment efficacy.

## Patients and Methods

A total of 57 treatment-experienced CHB patients at 2 medical centers who had failed or relapsed after previous antiviral therapy were enrolled in this study. The inclusion criteria included HBV DNA >20,000 IU/mL and alanine aminotransferase (ALT) levels between 1–10 fold the upper limit of normal (ULN) for the HBeAg-positive patients and HBV DNA >2000 IU/mL and ALT levels between 1–10-fold the ULN for the HBeAg-negative patients. The exclusion criteria included the following: HBV antiviral therapy within 6 months of enrollment; hepatitis C virus or human immunodeficiency virus co-infection; decompensated liver disease (a Child—Pugh score ≥ 7); pregnant or breast-feeding women; cytopenia (a white blood count <1500 cells/m^3^ and hemoglobin <12 mg/dL; a platelet count <90,000 cells/m^3^); evidence of alcoholism or drug abuse; and any other known disease for which Peg-IFN therapy was not suitable. The HBeAg-positive patients received Peg-IFN α-2a at180 μg weekly for 24 (as per reimbursement of the National Health Insurance of Taiwan) or 48 weeks (as per the patient’s willingness); the HBeAg-negative received the therapy for 48 weeks.

The liver biochemistry values, HBV serological markers, and HBV DNA, and HBsAg levels were tested before treatment and then every 3 months until 1 year after stopping the Peg-IFN treatment. HBeAg was detected by using commercially available enzyme-linked immunosorbent assay kits (Abbott Laboratories, North Chicago, IL, USA). The serum HBV DNA levels were determined using a CobasAmpliPrep/CobasTaqMan HBV assay (CAP/CTM, version 2.0, Roche Diagnostics, Indianapolis, IN, USA; the dynamic range was 20 IU/mL—1.7x10^8^IU/mL). The HBV genotype was determined using a restriction fragment length polymorphism (RFLP) method, as described earlier.[12] The serum HBsAg levels were quantified using an Architect HBsAg QT assay (Abbott Diagnostic, Germany). The sensitivity of the Architect assay ranged from 0.05 to 250 IU/mL. The samples with an HBsAg level higher than 250 IU/mL were further diluted at1: 500 and 1:1000 to obtain a reading within the range of the calibration curve.

The study was conducted according to the guidelines of the Declaration of Helsinki and the principles of Good Clinical Practice and was approved by the ethics committee of Kaohsiung Medical University Hospital. Written informed consent was obtained from all the patients.

The primary efficacy parameters were a combined response, defined as HBeAg seroconversion plus HBV DNA <2000 IU/mL 12 months post-treatment, in the HBeAg-positive patients and a virological response, defined as HBV DNA <2000 IU/mL 12 months post-treatment, in the HBeAg-negative patients. The secondary efficacy parameters included HBeAg seroconversion, HBV DNA <2000 IU/mL and HBsAg loss12 months post-treatment in the HBeAg-positive patients, and undetectable HBV DNA and HBsAg loss12 months post-treatment in the HBeAg-negative patients. The factors associated with primary treatment responses including the baseline biochemistry, baseline and on-treatment HBV DNA levels, and HBsAg levels were analyzed.

### Statistical analysis

Descriptive statistics were computed for all the variables, including the median as well as the 25th, and 75th percentiles for the continuous variables. For the categorical variables, the frequencies and percentages were estimated. The Mann-Whitney U test was used for comparing the continuous variables. The chi-square and Fisher’s exact tests were used for comparing the categorical variables. Binary logistic regression analysis was used to identify the independent associated factors. All the tests were two-sided, and a *P* value of <0.05 was considered statistically significant. All the analyses were performed using the SPSS 17.0 statistical package (SPSS, Inc., Chicago, IL, USA).

## Results

The baseline demographics of all the HBeAg-positive and -negative treatment-experienced CHB patients are shown in Table 1. Seventeen (29.8%) patients had undergone prior therapy with IFN. The 40 patients with prior NUC experience received lamivudine, including three patients who developed lamivudine resistant YMDD mutations. The treatment responses of the first course included the following: non-response (defined as no HBeAg loss at the end-of-treatment for the HBeAg-positive patients; HBV DNA > 2000 IU/mL at the end-of-treatment for the HBeAg-negative patients); partial response (defined as HBeAg loss without HBeAg seroconversion at the end-of-treatment for the HBeAg-positive patients and detectable HBV DNA but < 2000 IU/ml at the end-of-treatment for the HBeAg-negative patients); and relapse (defined as HBeAg seroconversion at the end-of-treatment for the HBeAg-positive patients and undetectable HBV DNA at the end-of-treatment for the HBeAg-negative patients) in 22 (38.6%), 4 (7.0%) and 31 (54.4%) of the patients, respectively. The genotype was determined in 55 patients, and approximately 60% of patients were genotype B. Most of the patients (70%) had an ALT level of greater than 2-fold ULN. The baseline HBV DNA levels were 6.6, 7.6, and 6.1 log_10_ IU/mL, and the baseline HBsAg levels were 3.4, 3.8, and 3.2 log_10_ IU/mL in all, HBeAg-positive, and -negative patients, respectively.

**Table 1:** Demographic data and baseline characteristics of all treatment-experienced CHB patients.

	Total(N = 57)	HBeAg-positive(N = 24)	HBeAg-negative(N = 33)
Male gender	45 (78.9)	15 (62.5)	30 (90.9)
Age, years	40.0 (31.5, 47.0)	34.0 (30.3, 40.5)	45.0 (38.5, 50.0)
Prior therapy			
IFN	17 (29.8)	8 (33.3)	9 (27.3)
NUC	40 (70.2)	16 (66.7)	24 (72.7)
Liver cirrhosis	3 (5.3)	0 (0)	3 (9.1)
Platelet, 10^3^/uL	180 (150, 208)	197 (157, 248)	174 (145, 197)
ALT, U/L	103.0 (71.5, 163.5)	112.0 (74.3, 171.3)	96.0 (66.0, 142.0)
ALT			
1-≤2x ULN	17 (29.8)	7 (29.2)	10 (30.3)
2 - 5x ULN	35 (61.4)	14 (58.3)	21 (63.6)
> 5x ULN	5 (8.8)	3 (12.5)	2 (6.1)
Albumin, g/dL	4.2 (4.1, 4.4)	4.1 (4.0, 4.3)	4.3 (4.2, 4.4)
Total bilirubin, mg/dL	1.0 (0.8, 1.3)	0.9 (0.8, 1.2)	1.0 (0.8, 1.3)
Creatinine, mg/dL	0.9 (0.7, 1.0)	0.9 (0.7, 0.9)	1.0 (0.8, 1.0)
Prothrombin time (INR)	1.04 (1.00, 1.12)	1.04 (1.00, 1.13)	1.04 (1.00, 1.10)
Genotype			
B	34/55 (61.8)	12/22 (54.5)	22 (66.7)
C	21/55 (38.2)	10/22 (45.5)	11 (33.3)
HBV DNA, log_10_ IU/mL	6.6 (5.8, 7.8)	7.6 (7.1, 8.1)	6.1 (5.0, 6.8)
HBsAg, log_10_ IU/mL	3.4 (2.9, 3.9)	3.8 (3.6, 4.4)	3.2 (2.3, 3.6)

Continuous variables: median (25th, 75th percentiles); categorical variables: numbers(percentages)

Missing data: genotype—2

IFN: interferon; NUC: nucleos(t)ide analogue; ALT: alanine aminotransferase; ULN: upper limit of normal; HBeAg: hepatitis B e antigen; HBsAg: hepatitis B surface antigen

### The efficacy of Peg-IFN α-2a in treatment experienced HBeAg-positive CHB patients

#### HBeAg seroconversion

**Fig 1:**
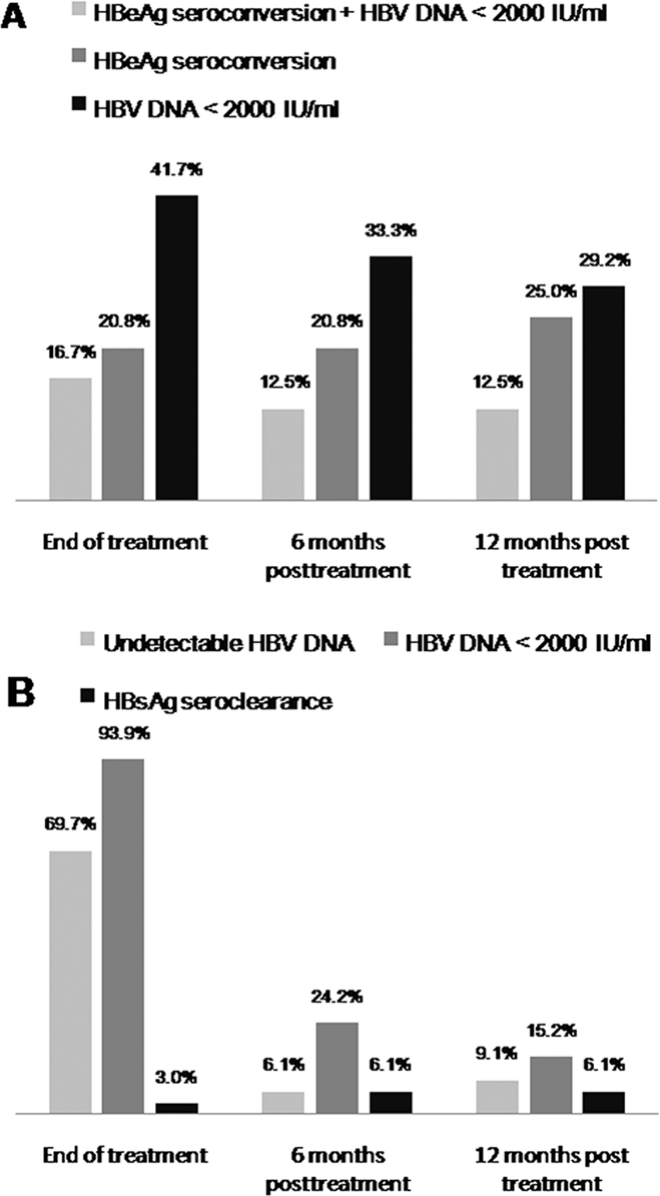
Response rates in HBeAg-positive and -negative patients.

The rates of HBeAg loss were 33.3% (8/24), 33.3% (8/24), and 33.3% (8/24) at the end of therapy, 6 months and 12 months post-treatment, respectively. The treatment responses of Peg-IFN therapy in the HBeAg-positive patients are shown in Fig. 1A. The HBeAg seroconversion rates were 20.8% (5/24), 20.8% (5/24), and 25.0% (6/24) at the end of therapy, 6 months post-treatment and 12 months post-treatment, respectively. The baseline high ALT level was the only significant factor associated with HBeAg seroconversion at 12 months off therapy (median [25^th^, 75^th^ percentile]: 177.5 [148–268] U/L vs. 88.5 [70.3–144.4] U/L, p = 0.009). Four of the 8 patients (50%) with prior IFN therapy achieved HBeAg seroconversion at 12 months off therapy compared to 2 of the 16 patients (12.5%) with prior NUC therapy (p = 0.129).

#### HBV virological response

The rates of achieving HBV DNA <2000 IU/mL were 41.7% (10/24), 33.3% (8/24), and 29.2% (7/24) at the end of therapy, 6 months post-treatment, and 12 months post-treatment, respectively. Three of the 10 HBeAg-positive patients who had HBV DNA <2000 IU/mL at the end of therapy experienced a virological relapse (HBV DNA >2000 IU/mL). One of the 3 patients who had an HBeAg seroconversion at end of treatment had an HBeAg seroreversion and clinical relapse (HBV DNA >2000 IU/mL ± ALT >2x ULN) 6 months after discontinuation of Peg-IFN therapy. Of two HBeAg-positive patients with a virological relapse, one developed HBeAg-negative CHB and one had persistent positive HBeAg. None of the three relapsers had an HBsAg loss. Prior IFN therapy (62.5%[5/8] vs. 12.5%[2/16] in prior NUC, p = 0.021), a baseline ALT level greater than 5-fold ULN (100.0% [3/3] vs. 19.0% [4/21], p = 0.017), and undetectable HBV DNA at 12 weeks of therapy (100.0% [3/3] vs. 19.0% [4/21], p = 0.017) were significant factors associated with HBV DNA <2000 IU/mL at 12 months off therapy.

#### Combined response

Of the patients, 16.7% (4/24), 12.5% (3/24), and 12.5% (3/24) achieved HBeAg seroconversion plus HBV DNA <2000 IU/mL at the end of therapy, 6 months post-treatment, and 12 months post-treatment, respectively. Female gender, prior IFN therapy, a baseline high ALT level, a low creatinine level, undetectable HBV DNA at 12 weeks and a decline in HBV DNA >2 log_10_ IU/mL at 12 weeks of therapy were significant factors associated with HBeAg seroconversion plus HBV DNA <2000 IU/mL at 12 months post-treatment (p = 0.042, 0.028, 0.040,0.041, 0.032 and 0.042, respectively). (Table 2)

**Table 2:** Factors associated with HBeAg seroconversion plus HBV DNA <2000 IU/mL 12 months post-treatment in treatment experienced HBeAg-positive CHB patients.

	HBeAg seroconversion plus HBV DNA < 2000 IU/ml		
	Yes (N = 3)	No (N = 21)	*P*
Female gender	3 (100)	6 (28.6)	0.042
Age, years	34.0 (31.0, 37.0)	34.0 (29.5, 41.0)	0.965
Prior IFN therapy	3 (100)	0 (0)	0.028
Platelet, 10^3^/uL	201 (165, 275)	193 (153, 245)	0.680
Baseline ALT, U/L	196.0 (157.0, 484.0)	98.0 (72.5, 165.0)	0.040
≥ 2x ULN (vs< 2x ULN)	3 (100)	14 (66.7)	0.530
≥5 x ULN (vs< 5x ULN)	1 (33.3)	2 (9.5)	0.343
Albumin, g/dL	3.9 (3.9, 4.3)	4.1 (4.0, 4.4)	0.354
Total bilirubin, mg/dL	1.2 (0.9, 1.4)	0.9 (0.7, 1.2)	0.354
Creatinine, mg/dL	0.7 (0.4, 0.7)	0.9 (0.7, 1.0)	0.041
Prothrombin time (INR)	1.16 (1.13, 1.19)	1.01 (0.99, 1.10)	0.052
Genotype B	1 (33.3)	11/19 (57.9)	0.571
Baseline HBV DNA, log_10_ IU/mL	7.9 (7.3, 8.1)	7.6 (7.0, 8.2)	0.600
Undetectable DNA at week 12	2 (66.7)	1 (4.8)	0.032
Decline in HBV DNA >2 log at week 12	3 (100)	6 (28.6)	0.042
Baseline HBsAg, log_10_ IU/mL	4.3 (3.4, 4.3)	3.8 (3.6, 4.4)	0.920
HBsAg < 1500 IU/mL at week 12	1 (33.3)	4 (19.0)	0.521
HBsAg > 20000 IU/mL at week 24	0 (0)	5 (23.8)	1.000
48 weeks Peg-IFN therapy	1 (33.3)	6 (28.6)	1.000

Continuous variables: median (25th, 75th percentiles); categorical variables: numbers(percentages)

IFN: interferon; ALT: alanine aminotransferase; ULN: upper limit of normal; HBeAg: hepatitis B e antigen; HBsAg: hepatitis B surface antigen

### Efficacy of Peg-IFN α-2a in treatment experienced HBeAg-negative CHB patients

At the end of therapy, 69.7% (23/33) and 93.9% (31/33) of the HBeAg-negative patients achieved undetectable HBV DNA levels and HBV DNA<2000 IU/mL, respectively. (Fig. 1B) The rates dramatically decreased to 6.1% (2/33) and 24.2% (8/33), respectively, at 6 months post-treatment and to 9.1% (3/33) and 15.2% (5/33), respectively, at 12 months post-treatment. Of the HBeAg-negative patients, 26 of the 31 patients who had HBV DNA < 2000 IU/mL at end of therapy experienced a virological relapse. Four of the 26 patients had a clinical relapse after discontinuation of the Peg-IFN therapy. No factor was significantly associated with HBV DNA <2000 IU/mL at 12 months off therapy. (Table 3) Undetectable HBV DNA levels at 12 weeks of therapy tended to be associated with undetectable HBV DNA at 12 months post-treatment (p = 0.067), and none of the 19 patients with detectable HBV DNA at 12 weeks of therapy achieved undetectable HBV DNA at 12 months post-treatment.

**Table 3:** Factors associated with HBV DNA <2000 IU/mL 12 months post-treatment in treatment experienced HBeAg-negative CHB patients.

	HBV DNA < 2000 IU/mL		
	Yes (N = 5)	No (N = 28)	*P*
Female gender	0 (0)	3 (10.7)	1.000
Age, years	40.0 (27.5, 48.0)	46.0 (39.0, 51.3)	0.200
Prior IFN therapy	1 (20)	8 (28.6)	1.000
Liver cirrhosis	1 (20)	2 (7.1)	0.400
Platelet, 10^3^/uL	174 (144, 204)	174 (144, 196)	0.802
Baseline ALT, U/L	107.0 (78.0, 217.0)	93.5 (64.5, 140.3)	0.393
≥ 2x ULN (vs< 2x ULN)	4 (80)	19 (67.9)	1.000
≥5 x ULN (vs< 5x ULN)	1 (20)	1 (3.6)	0.284
Albumin, g/dL	4.3 (4.0, 4.5)	4.3 (4.2, 4.4)	0.899
Total bilirubin, mg/dL	1.3 (0.8, 1.4)	1.0 (0.8, 1.3)	0.436
Creatinine, mg/dL	0.9 (0.9, 1.0)	1.0 (0.8, 1.1)	0.821
Prothrombin time (INR)	1.02 (0.97, 1.16)	1.05 (1.00, 1.10)	0.920
Genotype B	4 (80)	18 (64.3)	0.643
Baseline HBV DNA, log_10_ IU/mL	5.7 (5.0, 6.9)	6.1 (5.0, 6.9)	0.763
Undetectable DNA at week 12	4 (80)	10 (35.7)	0.138
Decline in HBV DNA >2 log at week 12	4 (80)	18 (64.3)	0.643
Undetectable DNA at week 24	4 (80)	9 (32.1)	0.066
Baseline HBsAg, log_10_ IU/mL	2.6 (2.3, 4.1)	3.2 (2.1, 3.6)	0.814
No HBsAg decline or HBV DNA decline < 2 log at week 12	2 (40)	10 (35.7)	1.000
HBsAg < 150 IU/mL at week 12	2 (40)	6 (21.4)	0.574
Decline in HBsAg ≥ 10% at week 24	5 (100)	19 (67.9)	0.290

Continuous variables: median (25th, 75th percentiles); categorical variables: numbers(percentages)

IFN: interferon; ALT: alanine aminotransferase; ULN: upper limit of normal; HBsAg: hepatitis B surface antigen

### On-treatment HBsAg level decline and off-treatment HBsAg loss

**Fig 2:**
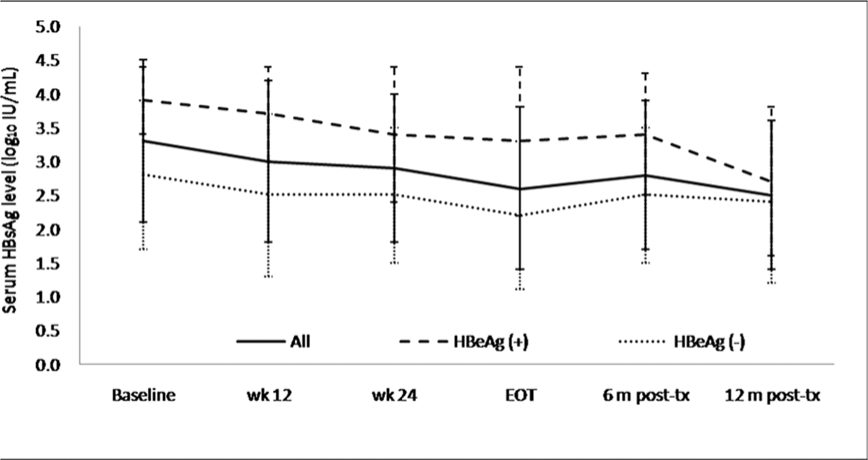
HBsAg kinetics during and after Peg-IFN therapy. The median HBsAg level from baseline to 12 months post-treatment declined from 3.4 to 2.6 log_10_ IU/mL in all the patients, from 3.8 to 2.7 log_10_ IU/mL in the HBeAg-positive patients, and from 3.2 to 2.5 log_10_ IU/mL in the HBeAg-negative patients.

**Fig 3:**
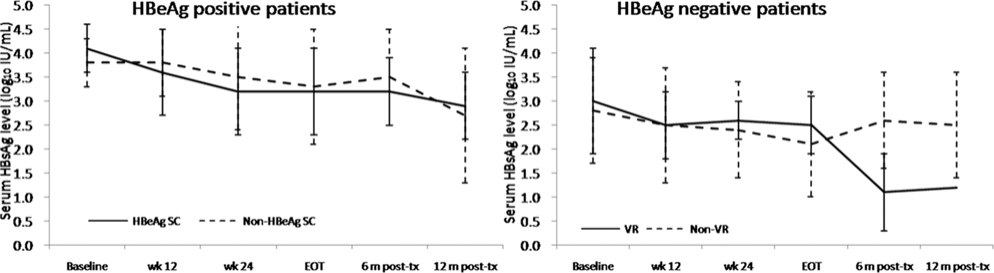
HBsAgkineticsduring and after Peg-IFN therapy according to the treatment response. There was no difference between the HBeAg-positive patients with and without HBeAgseroconversion in the HBsAg decline. The HBeAg-negative patients who achieved HBV DNA <2000 IU/mL at 12 months post-treatment showed continued HBsAg decline after stopping Peg-IFN. A rebound in the HBsAg level after stopping Peg-IFN was found in the HBeAg-negative patients who did not achieve HBV DNA <2000 IU/mL at 12 months post-treatment. (VR indicates HBV DNA <2000 IU/mL 12 months post-treatment)

The median HBsAg level from baseline to 12 months post-treatment declined from 3.4 to 2.6 log_10_ IU/mL in all the patients, from 3.8 to 2.7 log_10_ IU/mL in the HBeAg-positive patients, and from 3.2 to 2.5 log_10_ IU/mL in the HBeAg-negative patients. (Fig. 2) There was no difference in the HBsAg decline between the HBeAg-positive patients with and without HBeAg seroconversion. The HBeAg-negative patients who achieved HBV DNA <2000 IU/mL at 12 months post-treatment showed continued an HBsAg decline after stopping Peg-IFN. A rebound in the HBsAg level after stopping Peg-IFN was found in the HBeAg-negative patients who did not achieve an HBV DNA <2000 IU/mL level at 12 months post-treatment. (Fig. 3)

**Fig 4:**
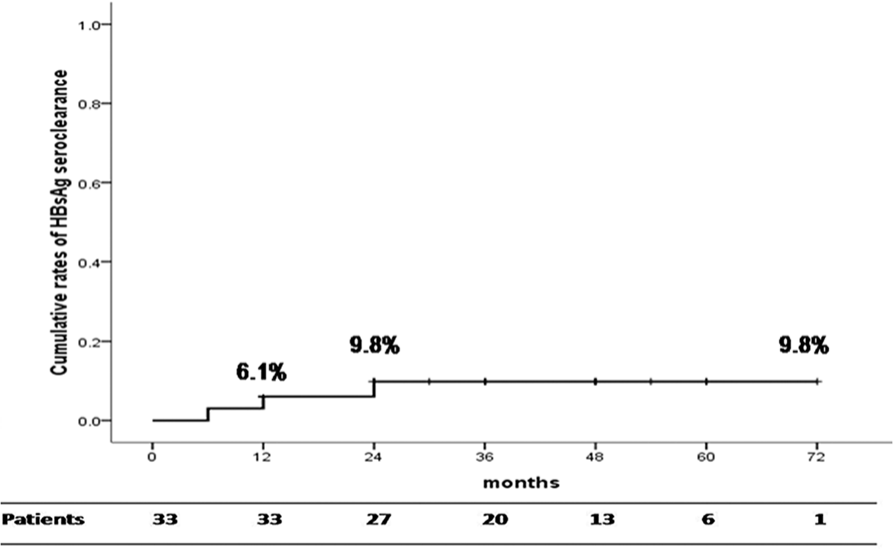
Cumulative rates of HBsAg loss in HBeAg-negative patients.

HBsAg loss was achieved in 1 (3.0%), 2 (6.1%), and 2 (6.1%) patients at the end of therapy, 6 months post-treatment, and 12 months post-treatment, respectively. (Fig. 1B) One of the patients with an HBsAg loss had seroconversion to anti-HBs. One patient who achieved an HBsAg loss 6 months post-treatment suffered an HBsAg reversion at 12 months post-treatment. Another patient had an HBsAg loss at 2 years post-treatment. For the HBeAg-negative patients with a median follow-up duration of 36 months (25^th^, 75^th^ percentiles: 18, 48 months), the 5-year cumulative HBsAg loss rate was 9.8%. (Fig. 4)

### Safety

None of the patients prematurely discontinued Peg-IFN α-2a therapy, and the dosage was not modified in any of the patients. Fifty-four patients (94.7%) completed 12 months of post-treatment follow-up. Three patients (5.3%) did not complete the follow-up because of liver decompensation related to a hepatitis B flare at 2, 6, and 7 months post-treatment, and they began NUC therapy immediately, with subsequent normalization of liver function.

## Discussion

This study demonstrated that Peg-IFN therapy for treatment-experienced CHB was effective for HBeAg-positive patients in terms of HBeAg seroconversion (25%) and the virological response (29%). Peg-IFN retreatment was unsatisfactory for combined serological and virological responses (12.5%) in HBeAg-positive patients and for the virological response (15%) in HBeAg-negative patients. Peg-IFN therapy was well tolerated in treatment-experienced CHB and might facilitate HBsAg loss in HBeAg-negative CHB patients.

The registration trial of Peg-IFN α-2a for HBeAg-positive CHB demonstrated that 32% and 14% of patients achieved HBeAg seroconversion and HBV DNA <400 copies/mL 6 months post-treatment, respectively.[7] A recent randomized control trial, the NEPTUNE study, further confirmed that 48 weeks of Peg-IFN α-2a at 180 μg/week is the most appropriate regimen, with the highest rates of HBeAg seroconversion, DNA suppression and combined response.[13] The data on Peg-IFN therapy in treatment-experienced CHB in the literature are limited. We demonstrated that 20.8%, 33.3% and 12.5% of treatment-experienced HBeAg-positive CHB patients achieved HBeAg seroconversion, DNA <2000 IU/mL, and a combined response 6 months off Peg-IFN retreatment, respectively. In the NEPTUNE study, 22.9% and 11.4% of the patients achieved HBeAg seroconversion and HBV DNA <2000 IU/mL, respectively, at 6 months off 24 weeks of Peg-IFN therapy, whereas these rates increased to 36.2% and 30%, respectively, with 48 weeks of therapy.[13] Approximately 70% of our HBeAg-positive patients received 24 weeks of Peg-IFN because of the reimbursement policy, which might be related in part to the lower response rates. The duration of therapy appeared to be unrelated to the treatment response in our study, although the small number of cases might limit the statistical analysis. The poor response was predominantly found in the patients with prior NUC therapy. Re-treatment with Peg-IFN achieved an equal or better response in the patients with prior IFN therapy than in the naïve patients. The causation of the relatively poor response in patients with prior NUC therapy requires further investigation.

Marcellin P. et al. reported the initial phase III study of Peg-IFN therapy in patients with HBeAg-negative CHB and demonstrated that 48 weeks of Peg-IFN Alfa-2a mono therapy achieved HBV DNA<400 and <20,000 copies/mL 24 weeks post-treatment in 19% and 43% of patients, respectively.[8] Of the Peg-IFN mono therapy group, 10% of the patients had undergone prior lamivudine (4%) or IFN alfa (6%) treatment. A long-term follow-up study showed that 31% and 5% of the patients achieved HBV DNA ≤2000 IU/mL and HBsAg clearance at 1 year post-treatment, respectively.[14] Our study showed an even lower rate of HBV DNA ≤2000 IU/mL (15.2%), with a similar HBsAg clearance rate (6.1%) in the treatment-experienced patients. The rapid re-appearance of HBV DNA after stopping Peg-IFN in the HBeAg-negative patients was observed in our study as well. The results suggest the need for a more stringent selection of treatment-experienced patients for retreatment with Peg-IFN and closer monitoring after stopping Peg-IFN.

A decline in HBV DNA >2 log_10_ IU/mL at week 12 was recently reported, which predicts treatment response in HBeAg-negative patients with Peg-IFN therapy.[15] In our study, the results showed that a decline in HBV DNA >2 log_10_ IU/mL at week 12 is significantly associated with the treatment response in the HBeAg-positive patients (p = 0.042) and not in the HBeAg-negative patients (p = 0.643). Further study is needed to clarify the predictive value of an on-treatment decline in HBV DNA in HBeAg-positive and -negative patients.

Serum HBsAg levels have been associated with treatment response to Peg-IFN therapy. On-treatment declines in HBsAg level could predict treatment response or failure in HBeAg-positive patients.[16,17] Further studies have identified the positive predictive role of an HBsAg level <1500 IU/mL at week 12 and a negative predictive role of an HBsAg level >20,000 IU/mL at week 24.[13,18–20] For HBeAg-negative patients, an on-treatment HBsAg decline of <0.5 log at week 12, of <10% at week 24, and an absence of any decline in the HBsAg level with a <2 log decline in the HBV DNA level at week 12 were reported as negative predictors of HBV DNA suppression and/or HBsAg loss after stopping Peg-IFN therapy.[20–22] For HBeAg-negative patients, one study from Italy demonstrated that HBsAg at the end of the treatment could predict the long-term virological response.[23] In this study, the patients with a virological response had even higher HBsAg levels at the end of treatment compared to those without a virological response. They had, instead, a significant decline after discontinuation of PegIFN therapy. This result might be a novel finding for Eastern patients and require a large-scale study for confirmation. Additionally, our recent study demonstrated that a serum HBsAg cut-off of 150 IU/mL at week 12 strongly predicts a sustained response with HBV DNA <312 copies/mL at 24 weeks off Peg-IFN therapy in HBeAg-negative CHB patients infected with genotype B or C.[24] The same predictive effect for the HBsAg level was not shown in this study although the genotype distribution and racial population were identical, possibly because of the small number of patients. The role of the HBsAg level in the prediction of a response to Peg-IFN therapy in treatment-experienced CHB patients remains to be studied.

None of our patients developed HCC during the follow up period, regardless of whether the treatment failed or succeeded. The patients who failed the Peg-IFN therapy might be more prone to developing HCC in succeeding years and should have a strict follow-up.

One limitation of this study was its relatively small sample size. Whether our results could be extrapolated to treatment-experienced patients in general remains to be studied. Another limitation of this study was that a major proportion of the HBeAg-positive patients received only 24 weeks of Peg-IFN therapy, which might have a negative effect on the response rate. A prospective study with a larger cohort of patients treated with 48 weeks of Peg-IFN is warranted. Given the unmet need for the retreatment of CHB patients and the limited literature data, our results provide important information regarding the role of finite therapy with Peg-IFN in treatment-experienced patients.

The retreatment of CHB with Peg-IFN was effective for HBeAg-positive patients in terms of HBeAg seroconversion and the virological response, particularly for patients with prior IFN treatment experience. Although the virological response to the re-treatment of HBeAg-negative patients was unsatisfactory, Peg-IFN might facilitate HBsAg loss in the clinical setting.
